# ESG performance, financing constraints, and workplace health investment: evidence from Chinese biopharmaceutical enterprises

**DOI:** 10.3389/fpubh.2026.1765422

**Published:** 2026-03-09

**Authors:** Aimin Li, Shuyu Zhou

**Affiliations:** Guangzhou Huashang College, Guangzhou, China

**Keywords:** biopharmaceutical industry, China, ESG performance, financing constraints, occupational health, workplace health promotion

## Abstract

**Background:**

Workplace health promotion (WHP) is critical for occupational health management, yet empirical evidence on determinants of corporate health investment remains limited. This study examines whether ESG performance promotes workplace health investment in Chinese biopharmaceutical enterprises and investigates the mediating role of financing constraints.

**Methods:**

Using panel data from 55 A-share listed biopharmaceutical companies (2015–2023, 495 firm-year observations), we employed fixed effects models with manually collected health expenditure data. The three-step method tested financing constraints as a mediator. Heterogeneity analyses and multiple endogeneity treatments were conducted.

**Results:**

ESG performance significantly promotes workplace health investment (*β* = 0.0024, *p* > 0.01), with financing constraints mediating 20.8% of the total effect. The Social dimension shows the strongest association. Effects are stronger in non-state-owned enterprises, high R&D intensity firms, and vaccine/blood products companies. Health expenditure reduces R&D personnel turnover and enhances innovation output.

**Conclusion:**

ESG performance effectively promotes workplace health investment by alleviating financing constraints, providing empirical support for integrating occupational health promotion into corporate sustainability strategies. Theoretically, this study bridges corporate finance and occupational health research by revealing the transmission mechanism of “ESG → financing constraint alleviation → workplace health expenditure.” Practically, the findings suggest that ESG improvement can serve as a market-based lever for promoting organizational investment in employee health programs.

## 1 Introduction

Workplace health promotion (WHP) has become a cornerstone of modern occupational health management. The World Health Organization defines a healthy workplace as one where workers and managers collaborate to use a continual improvement process to protect and promote the health, safety, and well-being of all workers. The International Labour Organization’s Promotional Framework for Occupational Safety and Health Convention (C187) emphasizes that occupational health should be integrated into broader organizational management systems. Despite these global frameworks, translating occupational health principles into corporate investment decisions remains a significant challenge, particularly in industries characterized by complex occupational hazards.

The biopharmaceutical industry presents a compelling case for examining workplace health investment. Research and development personnel in this sector face a unique constellation of occupational health risks: exposure to chemical reagents and solvents during drug synthesis, handling of biological agents and potentially infectious materials, radiation exposure in certain testing procedures, and significant psychosocial stressors including extended working hours, tight project deadlines, and the psychological burden of lengthy and uncertain drug development cycles. These occupational hazards make workplace health promotion particularly critical in this industry, yet research on how corporate governance factors influence occupational health investment remains scarce.

Public health serves as a fundamental cornerstone of national development. In 2016, China elevated national health to a strategic priority through the “Healthy China 2030” Planning Outline, explicitly proposing the core concept of “integrating health into all policies.” Recent empirical evidence confirms that this policy has significantly improved population health outcomes (1). The biopharmaceutical industry, as a crucial pillar supporting the Healthy China strategy, bears the critical mission of ensuring pharmaceutical supply and advancing medical innovation. The global COVID-19 pandemic has further highlighted the public health value of biopharmaceutical enterprises while imposing higher requirements on their sustainable development capabilities and workforce health management.

Concurrently, ESG (Environmental, Social, and Governance) investment philosophy has witnessed rapid global expansion. According to the Global Sustainable Investment Alliance, global ESG assets under management exceeded 30 trillion USD in 2022. China’s capital market has also accelerated ESG practices, with the China Securities Regulatory Commission issuing the “Guidelines for Listed Companies’ Investor Relations Management” in 2022, incorporating ESG information into investor relations management for the first time. For biopharmaceutical enterprises, ESG represents not merely an external compliance requirement but a strategic choice for enhancing sustainable competitiveness. Importantly, occupational health and safety constitute core issues within the “Social” dimension of the ESG framework, creating a natural linkage between ESG performance and workplace health investment.

Biopharmaceutical firms are characterized by lengthy R&D cycles and high talent dependency, where the physical and mental health of R&D personnel directly affects the quality and continuity of innovation output. Studies on Chinese pharmaceutical listed companies have demonstrated that ESG performance significantly promotes corporate innovation (2). However, corporate health investment often faces funding constraints—when financing conditions tighten, “non-rigid” expenditures such as workplace health promotion are typically the first to be curtailed. Can ESG performance promote corporate health investment by improving the financing environment? This question carries both theoretical and practical significance for occupational health promotion.

Accordingly, this study focuses on the following core research questions: Does ESG performance promote workplace health investment in biopharmaceutical enterprises? Does financing constraint play a mediating role between ESG and health expenditure? Does the effect of ESG on health expenditure exhibit heterogeneity across enterprises with different ownership types, R&D intensities, and industry sub-sectors? How does workplace health investment subsequently affect occupational outcomes such as employee retention and productivity?

Prior research has explored the relationship between ESG and employee-related outcomes from various perspectives. Studies have demonstrated that CSR activities positively influence employee satisfaction and organizational commitment (3), and that employee-friendly firms exhibit higher labor investment efficiency (4). In the occupational health domain, systematic reviews have examined the linkage between CSR and employee health and well-being, calling for more research on organizational-level determinants (5, 6). Regarding ESG and corporate resource allocation, existing literature has primarily focused on R&D investment, capital expenditure, and green innovation, confirming that ESG performance influences corporate investment decisions through financing channels (7, 8). However, several gaps remain in the literature. First, while studies have examined ESG’s effects on aggregate employee welfare, few have specifically investigated workplace health expenditure as a distinct resource allocation decision. Second, the transmission mechanism through which ESG affects health investment—particularly the role of financing constraints—has not been empirically tested. Third, industry-specific evidence from high-risk sectors such as biopharmaceuticals, where occupational health management is particularly critical, remains scarce. This study aims to address these gaps by providing direct empirical evidence on the ESG-health investment relationship and its underlying mechanism.

This study employs a sample of 55 A-share listed biopharmaceutical companies in China from 2015 to 2023, utilizing fixed effects models to examine the impact of ESG performance on workplace health expenditure. Health expenditure data were manually collected from corporate annual reports, encompassing medical insurance, occupational health protection expenditures, health examinations, occupational health training, and Employee Assistance Programs. Building upon the baseline regression, this study applies the three-step method to test the mediating effect of financing constraints and conducts heterogeneity analysis from three dimensions: ownership type, R&D intensity, and industry sub-sector.

The contributions of this study to occupational health research are threefold: First, it bridges corporate finance and occupational health research by demonstrating how ESG performance—a market-based governance mechanism—can serve as a lever for promoting workplace health investment. Second, it uncovers the transmission mechanism of “ESG → financing constraint alleviation → workplace health expenditure” and provides an in-depth analysis of three specific channels: information, cost of capital, and stakeholder. Third, it provides empirical evidence on the occupational health-productivity linkage, showing that workplace health investment reduces R&D personnel turnover and enhances innovation output, thereby offering evidence-based support for occupational health practitioners advocating for increased organizational investment in employee health programs.

## 2 Literature review and hypotheses development

### 2.1 Workplace health promotion: concepts and determinants

Workplace health promotion (WHP) encompasses the combined efforts of employers, employees, and society to improve the health and well-being of people at work. The Luxembourg Declaration on Workplace Health Promotion defines WHP as combining: (1) improving work organization and the working environment; (2) promoting active participation; and (3) encouraging personal development. The WHO Healthy Workplace Framework emphasizes that healthy workplaces should address physical work environment, psychosocial work environment, personal health resources, and enterprise community involvement.

From an occupational health perspective, WHP programs have gained increasing attention as effective interventions for improving employee health outcomes and reducing occupational diseases. The biopharmaceutical industry presents unique occupational health challenges that make WHP particularly salient. R&D personnel face exposure to chemical hazards (solvents, reagents, intermediates), biological hazards (cell cultures, viral vectors, recombinant proteins), physical hazards (noise, radiation), and significant psychosocial stressors (deadline pressure, job insecurity during lengthy development cycles, irregular working hours). These workplace exposures necessitate comprehensive occupational health management approaches.

Research has demonstrated that comprehensive WHP programs can reduce absenteeism, improve productivity, and enhance employee retention. Recent evidence from Frontiers in Public Health confirms that organizational health-oriented strategies significantly improve employees’ job performance, with psychological wellbeing serving as a key mediating mechanism (9). A systematic review examining CSR and internal stakeholders’ health and well-being provides important conceptual foundations for understanding these relationships (5). Studies have shown that CSR and workplace health promotion show positive reciprocal effects, requiring committed leadership and integration into sustainability policies (6). However, the relationship between corporate governance mechanisms, particularly ESG performance, and occupational health investment has not been systematically examined.

Corporate health expenditure refers to various investments made by enterprises to maintain and promote employee physical and mental health, including medical insurance, occupational health protection, health examinations, health training, and employee assistance programs. The existing literature explores the determinants of health expenditure from three levels: firm characteristics, governance structure, and external environment.

At the firm characteristics level, larger firms with stronger profitability typically exhibit higher health expenditure levels (10). R&D-intensive firms, given their high dependence on human capital, are more inclined to increase health investment to maintain talent competitiveness (11). Regarding governance structure, board diversity and independence are positively associated with employee welfare expenditure (12). Evidence shows that employee-friendly firms invest more efficiently, particularly in labor investment (4). At the external environment level, labor market competition intensity and social responsibility pressures drive enterprises to increase health investment.

Notably, financing constraints represent a significant factor limiting corporate health expenditure. Health investment is characterized by long payback periods and uncertain returns; when enterprises face financing difficulties, they often prioritize cutting such “non-rigid” expenditures (13). Therefore, alleviating financing constraints may represent an effective pathway for promoting corporate health expenditure.

### 2.2 Conceptual distinction between ESG performance and workplace health investment

Before developing our hypotheses, it is essential to address a potential conceptual concern: workplace health-related elements may be incorporated as components of the “Social” pillar in ESG rating methodologies, raising questions about whether our analysis risks tautological inference. We argue that ESG performance and workplace health investment (WHP) are conceptually and empirically distinct constructs, and we take multiple steps to ensure credible causal identification.

First, there is a fundamental measurement distinction between ESG scores and WHP investment. ESG performance, as measured by third-party rating agencies such as SynTao Green Finance, represents an external assessment based on corporate disclosures and standardized evaluation criteria. The SynTao ESG system encompasses 14 themes and 26 key indicators across environmental, social, and governance dimensions, of which employee health-related indicators constitute only a small subset within the Social pillar—primarily assessing policy existence, management systems, and disclosure quality rather than actual expenditure levels. In contrast, our WHP investment measure captures actual financial expenditure on employee health, manually extracted from annual reports including medical insurance payments, occupational health protection costs, health examination fees, and EAP expenditures. This distinction is crucial: ESG scores reflect perceived performance based on external assessments of corporate practices and policies, while WHP investment reflects actual internal resource allocation decisions measured in monetary terms.

Second, we explicitly map three potential causal pathways to distinguish our hypothesized mechanism from alternative explanations:

(1) ESG → Financing constraints → WHP (Primary hypothesis): Superior ESG performance reduces financing constraints through information, cost of capital, and stakeholder channels, which relaxes financial barriers and enables greater WHP investment. This market-based mechanism operates independently of any mechanical correlation in ESG scoring.
(2) WHP → Improved ESG (Reverse causality): Increased WHP investment may subsequently improve ESG scores through enhanced social performance disclosure. We address this through one-year and two-year lagged ESG variables, establishing temporal precedence.
(3) Common factors → Both (Omitted variable bias): Unobserved firm characteristics (e.g., managerial quality, corporate culture) may simultaneously drive both ESG performance and WHP investment. We control for this through firm fixed effects and comprehensive firm-level controls.

Third, to directly test whether our results are driven by mechanical correlation within the Social pillar, we conduct a decomposition analysis using only the Environmental (E) and Governance (G) dimensions, excluding the Social (S) dimension entirely. If our findings persist when using only E and G components—which have no direct connection to employee health metrics—this would provide strong evidence that the ESG-WHP relationship reflects genuine resource effects rather than measurement overlap.

### 2.3 Economic consequences of ESG performance

ESG (Environmental, Social, Governance), as a comprehensive framework for measuring corporate sustainable development capabilities, has become a focal point in academic research regarding its economic consequences. A comprehensive review of ESG and CSR research in corporate finance provides foundational frameworks for understanding these relationships (14). The existing literature primarily examines three dimensions: financial performance, firm value, and financing capability.

Regarding financial performance, a meta-analysis of over 2,000 studies found that approximately 90% supported a non-negative relationship between ESG and financial performance, with the majority demonstrating a positive correlation (15). Further research indicated that ESG strengths can enhance firm value, while ESG weaknesses produce significant negative effects (16). Research using stakeholder theory frameworks has examined how ESG dimensions individually relate to performance in emerging markets (17). In the Chinese context, ESG performance exerts a significant positive influence on corporate performance, with this effect being more pronounced in non-state-owned enterprises (18). Evidence from Chinese A-share listed companies further confirms the positive relationship between ESG activities and firm performance, with notable variations across ownership types (19).

Concerning financing capability, using a cross-national sample from 49 countries, researchers found that firms with superior ESG performance face lower financing constraints, primarily attributable to ESG’s reduction of agency costs and information asymmetry (7). Studies have demonstrated that high-ESG firms enjoy lower equity financing costs (20). Recent evidence from China shows that ESG performance significantly reduces the cost of equity capital through mechanisms of reduced market risk and increased equity diversification (21). Chinese scholars have confirmed that ESG performance can significantly reduce corporate debt financing costs (22).

However, existing research predominantly focuses on ESG’s impact on external stakeholders (shareholders, creditors), with relatively insufficient attention to internal stakeholders, particularly employees. Emerging research has begun to explore CSR’s influence on employee-level outcomes, demonstrating that CSR practices can reduce employee burnout through improved subjective wellbeing and compassion (23), and promote safety behavior through enhanced psychological safety (24). As carriers of core human capital, employees’ health and well-being directly influence corporate sustainable development capabilities. This study extends the research perspective to employee health investment, exploring ESG’s impact on workplace health promotion expenditure.

### 2.4 The mediating role of financing constraints

Financing constraint theory posits that due to information asymmetry and agency problems, the cost of external financing exceeds that of internal financing, causing investment decisions to be constrained by fund availability (25). ESG performance may alleviate financing constraints through the following mechanisms:

First, the information mechanism. ESG disclosure reduces information asymmetry between firms and external investors, enhances corporate transparency, and enables investors to more accurately assess firm risk and value (26). Research on Chinese listed companies demonstrates that ESG information disclosure significantly reduces stock price crash risk through alleviating information asymmetry and enhancing corporate reputation (27). Furthermore, ESG disclosure quality moderates the relationship between ESG performance and firm value, highlighting the importance of transparency (28).

Second, the signaling mechanism. High ESG performance signals firm quality to the market, indicating that management possesses long-term orientation and risk management capabilities, thereby strengthening investor confidence (29).

Third, the stakeholder mechanism. ESG performance improvement facilitates the establishment of favorable relationships with various stakeholders, reduces transaction costs and operational risks, and consequently secures better financing conditions (30).

Empirical evidence from China confirms that ESG performance directly reduces financing constraints and attracts institutional investors, with effects being more significant in non-state-owned enterprises (8). Using staggered difference-in-differences methodology, researchers have found that ESG ratings reduce cost of capital through reduced operational risks, with effects more pronounced in non-state-owned and highly competitive enterprises (31). Once financing constraints are alleviated, firms possess more abundant financial resources for various investment activities, including workplace health promotion expenditure.

### 2.5 Hypotheses development

#### 2.5.1 ESG performance and workplace health investment (H1)

Based on the above analysis, ESG performance may promote corporate health expenditure through multiple pathways. From the resource effect perspective, ESG releases financial resources by alleviating financing constraints, providing the material foundation for health investment. From the ideology effect perspective, high-ESG firms typically possess stronger social responsibility awareness and stakeholder orientation, placing greater emphasis on employee health and well-being—a core component of occupational health management. From the reputation effect perspective, health investment helps maintain and enhance corporate ESG image, forming a positive feedback loop. Accordingly, this study proposes:

*H*1: ESG performance positively promotes workplace health investment in biopharmaceutical enterprises.

#### 2.5.2 The mediating role of financing constraints (H2)

The promoting effect of ESG on health expenditure may be partially realized through the alleviation of financing constraints. On one hand, ESG performance improvement reduces information asymmetry and financing costs, thereby alleviating corporate financing constraints; on the other hand, the alleviation of financing constraints releases financial resources, enabling firms to increase workplace health expenditure. This transmission path of “ESG → financing constraint alleviation → health expenditure” constitutes a mediating effect. Accordingly, this study proposes:

*H*2: Financing constraints play a mediating role between ESG performance and workplace health investment.

#### 2.5.3 The moderating effect of ownership type (H3)

Compared to state-owned enterprises (SOEs), non-state-owned enterprises (non-SOEs) typically face more severe financing constraints (32). The “ownership discrimination” in bank credit resource allocation makes it more difficult for non-SOEs to obtain low-cost funding. Therefore, ESG performance improvement generates greater marginal value in alleviating financing constraints for non-SOEs, which subsequently translates into more significant increases in health expenditure. Additionally, non-SOEs possess more flexible decision-making mechanisms, enabling faster responses to resource release resulting from ESG improvement. Accordingly, this study proposes:

*H*3: Compared to SOEs, the promoting effect of ESG on workplace health investment is more significant in non-SOEs.

#### 2.5.4 The moderating effect of R&D intensity (H4)

High R&D intensity firms exhibit greater dependence on human capital. R&D personnel represent the most critical strategic resource for biopharmaceutical enterprises, and their health status directly influences the quality and continuity of innovation output. From an occupational health perspective, high R&D intensity implies greater exposure to workplace hazards and psychosocial stressors, making workplace health promotion more imperative. Consequently, high R&D firms possess stronger motivation to translate ESG improvement into employee health investment to maintain talent competitive advantage. Research across 30 countries demonstrates that employee satisfaction as a non-financial performance metric translates into tangible corporate financial outcomes (33). Simultaneously, the marginal returns on employee health investment are higher in high R&D firms, providing stronger investment incentives. Accordingly, this study proposes:

*H*4: Compared to low R&D intensity firms, the promoting effect of ESG on workplace health investment is more significant in high R&D intensity firms.

#### 2.5.5 The moderating effect of industry sub-sector (H5)

Significant sub-sector differences exist within the biopharmaceutical industry regarding occupational health risk profiles. Vaccine and blood products manufacturing involves biosafety risks, handling of potentially infectious materials, and stringent regulatory requirements for occupational health management; these firms have established relatively comprehensive occupational health management systems, facilitating the translation of ESG improvement into standardized health investment. In contrast, innovative drug companies face high R&D uncertainty, with funding predominantly directed toward R&D pipelines, resulting in relatively lower priority for health expenditure. Accordingly, this study proposes:

*H*5: Compared to innovative drug companies, the promoting effect of ESG on workplace health investment is more significant in vaccine/blood products companies.

**Figure 1:**
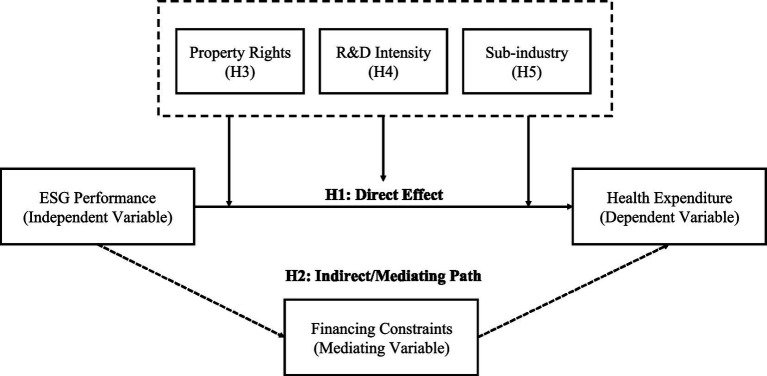
Research framework and hypotheses. Solid arrows indicate direct effects, dashed arrows indicate mediating paths, and boxes represent moderating factors.

The research framework and hypothesis relationships of this study are illustrated in Figure 1.

## 3 Research design

### 3.1 Sample selection and data sources

#### 3.1.1 Sample selection

This study focuses on A-share listed biopharmaceutical companies in China, with the sample period spanning from 2015 to 2023. The selection of 2015 as the starting year is primarily based on the following considerations: first, SynTao Green Finance ESG rating data has provided relatively comprehensive coverage since 2015; second, ESG information disclosure has become progressively standardized after 2015, ensuring better data availability and comparability.

The sample selection process proceeded as follows: (1) using the CSRC industry classification of “pharmaceutical manufacturing” as the foundation, combined with Wind industry classification, biopharmaceutical-related enterprises were identified; (2) ST, *ST, and delisted companies were excluded; (3) companies whose primary business consists of medical devices, pharmaceutical distribution, or CRO/CDMO services rather than biopharmaceutical manufacturing were excluded; (4) companies with substantial missing data for key variables were excluded; (5) companies listed for less than 1 year were excluded. The final sample comprises 55 biopharmaceutical enterprises, encompassing four sub-sectors: vaccines (8 firms), blood products (6 firms), other biological products (15 firms), and innovative drugs (26 firms). The complete list of sample companies is presented in Appendix Table A. Using firm-year as the unit of observation, approximately 495 observations were obtained.

#### 3.1.2 Data sources

Data sources for this study are as follows: (1) ESG rating data were obtained from the SynTao Green Finance ESG rating database, with Wind ESG and Sino-Securities ESG ratings used in robustness tests from their respective databases; (2) financial data and corporate governance data were sourced from the CSMAR database; (3) workplace health expenditure data were manually collected from corporate annual reports, primarily extracted from the “Employee Compensation Payable” notes, social responsibility reports, and ESG reports containing employee health-related disclosures. All continuous variables were winsorized at the 1st and 99th percentiles to eliminate the influence of extreme values.

### 3.2 Variable definitions

#### 3.2.1 Dependent variable: workplace health expenditure

This study manually extracted employee health-related expenditure data from corporate annual reports to construct the workplace health expenditure indicator. Following the WHO Healthy Workplace Framework and occupational health literature, the specific components of health expenditure include: (1) medical insurance and supplementary medical insurance expenses—basic health coverage ensuring access to healthcare services; (2) occupational health protection expenditures within safety production expenses—investments specifically targeting workplace hazard mitigation; (3) employee health examination expenses—including both general health screenings and occupation-specific medical surveillance (e.g., biomonitoring for chemical exposures); (4) occupational health training expenses—programs educating employees about workplace hazards and health-protective behaviors; (5) Employee Assistance Program (EAP) expenditures—mental health support and counseling services addressing psychosocial aspects of occupational health.

Three measurement approaches were employed for health expenditure: (1) health expenditure intensity (HE_Int), calculated as total health expenditure divided by operating revenue, reflecting the relative level of corporate health investment; (2) per capita health expenditure (HE_Per), calculated as total health expenditure divided by the number of employees, reflecting the health investment level at the individual employee level; (3) natural logarithm of health expenditure (Ln_HE), calculated as ln(total health expenditure + 1), used for robustness tests. The baseline regression employs health expenditure intensity (HE_Int) as the primary dependent variable.

#### 3.2.2 Independent variable: ESG performance

This study employs SynTao Green Finance ESG ratings as the proxy variable for ESG performance. The SynTao ESG rating system encompasses three dimensions: Environmental (E), Social (S), and Governance (G), comprising 14 themes and 26 key indicators. The rating results are classified into nine levels (C to AAA), which this study converts into a continuous variable ranging from 1 to 9 (C = 1, CC = 2, CCC = 3, B = 4, BB = 5, BBB = 6, A = 7, AA = 8, AAA = 9). The selection of SynTao ESG is based on its extensive coverage, high update frequency (quarterly updates), and localized adjustments tailored to the Chinese market context.

To examine the differentiated effects of ESG dimensions, this study further extracts the Environmental score (E_Score), Social score (S_Score), and Governance score (G_Score) for dimension-specific analysis. The Social dimension is particularly relevant from an occupational health perspective as it encompasses employee-related indicators including occupational health and safety practices. In robustness tests, Wind ESG ratings, Sino-Securities ESG ratings, and an ESG report disclosure dummy variable are employed as alternative indicators.

#### 3.2.3 Mediating variable: financing constraints (SA)

This study employs the SA index as the proxy variable for financing constraints. The SA index, developed by Hadlock and Pierce (35), is calculated as:


$$\mathrm{SA}=-0.737\times \text{Size}+0.043\times {\text{Size}}^{2}–0.040\times \mathrm{Age}$$

where Size represents the natural logarithm of total assets, and Age represents the firm’s listing age. The SA index yields negative values, with larger absolute values indicating more severe financing constraints. This study uses the absolute value of the SA index in empirical analysis to facilitate coefficient interpretation. The selection of the SA index is based on its inclusion of only two exogenous variables—firm size and age—effectively avoiding endogeneity issues, and its extensive validation in the Chinese context. In robustness tests, the KZ index and WW index are employed as alternative indicators.

While the SA index offers significant advantages in terms of exogeneity, we acknowledge certain limitations inherent in its construction. First, by relying solely on firm size and age, the SA index may not fully capture the multidimensional nature of financing constraints, such as firms’ cash flow conditions, collateral availability, or banking relationships. Second, the assumption that smaller and younger firms face greater financing constraints, while generally valid, may not hold uniformly across all industry contexts—particularly in the biopharmaceutical sector, where small innovative firms may attract venture capital or policy support. Third, the SA index cannot capture time-varying changes in financing constraints driven by macroeconomic conditions or firm-specific events. To mitigate these limitations, we employ the KZ index and WW index as alternative measures in robustness tests, both of which incorporate additional financial variables (e.g., cash flow, leverage, dividends). The consistency of results across different financing constraint measures enhances confidence in our findings.

#### 3.2.4 Control variables

Following prior literature, this study controls for the following factors that may affect corporate health expenditure: (1) firm size (Size), measured by the natural logarithm of total assets; (2) leverage (Lev), measured by total liabilities divided by total assets; (3) profitability (ROA), measured by net income divided by total assets; (4) revenue growth rate (Growth), measured by the growth rate of operating revenue; (5) cash flow (CF), measured by net operating cash flow divided by total assets; (6) Tobin’s Q (TobinQ), measured by market value divided by total assets; (7) ownership concentration (Top1), measured by the shareholding ratio of the largest shareholder; (8) board size (Board), measured by the natural logarithm of the number of board members; (9) independent director ratio (Indep), measured by the number of independent directors divided by board size; (10) CEO duality (Dual), equals 1 if the chairman and CEO positions are held by the same person, 0 otherwise; (11) ownership type (SOE), equals 1 for state-owned enterprises, 0 otherwise; (12) R&D intensity (RD_Int), measured by R&D expenditure divided by operating revenue; (13) firm age (Age), measured by listing years.

The main variable definitions are summarized in Table 1.

**Table 1:** Variable definitions and descriptive statistics.

Variable	Definition	N	Mean	S. D.	Min	Median	Max
HE_Int	Health expenditure / Operating revenue	495	0.021	0.015	0.005	0.018	0.078
HE_Per	Health expenditure / Number of employees (10,000 CNY)	495	2.58	1.42	0.56	2.31	8.65
Ln_HE	ln(Health expenditure + 1)	495	17.24	1.08	14.62	17.15	20.31
ESG	SynTao ESG rating (1–9)	495	5.42	1.18	2.00	5.50	8.00
E_Score	Environmental dimension score	495	5.18	1.35	1.50	5.00	8.50
S_Score	Social dimension score	495	5.56	1.22	2.00	5.50	8.00
G_Score	Governance dimension score	495	5.51	1.08	2.50	5.50	8.00
SA	SA index (absolute value)	495	3.82	0.31	3.15	3.79	4.68
Size	ln(Total assets)	495	22.48	1.15	20.12	22.38	25.86
Lev	Total liabilities / Total assets	495	0.28	0.14	0.05	0.26	0.65
ROA	Net income / Total assets	495	0.08	0.07	−0.15	0.07	0.32
Growth	Operating revenue growth rate	495	0.18	0.35	−0.42	0.12	2.15
CF	Operating cash flow / Total assets	495	0.07	0.08	−0.18	0.06	0.35
TobinQ	Market value / Total assets	495	3.56	2.41	0.85	2.89	15.23
Top1	Largest shareholder ownership ratio	495	0.32	0.13	0.09	0.30	0.68
Board	ln(Number of board members)	495	2.12	0.21	1.61	2.08	2.71
Indep	Independent directors / Board size	495	0.38	0.05	0.33	0.36	0.57
Dual	CEO duality = 1	495	0.35	0.48	0	0	1
SOE	State-owned enterprise = 1	495	0.18	0.38	0	0	1
RD_Int	R&D expenditure / Operating revenue	495	0.12	0.08	0.02	0.10	0.45
Age	Listing years	495	12.35	6.28	1	12	28

### 3.3 Model specification

#### 3.3.1 Baseline model

To test Hypothesis 1, this study constructs the following fixed effects model:


$$\mathrm{HE}\_{\mathrm{Int}}_{\mathrm{it}}=\beta_{0}+\beta_{1}{\mathrm{ESG}}_{\mathrm{it}}+\beta_{2}{\text{Controls}}_{\mathrm{it}}+\mu_{i}+\lambda_{t}+\varepsilon_{\mathrm{it}}$$

where i denotes the firm, t denotes the year, HE_Int represents workplace health expenditure intensity, ESG represents ESG performance, Controls represents control variables, *μ* represents firm fixed effects, *λ* represents year fixed effects, and *ε* represents the error term. A significantly positive *β*₁ would indicate that ESG performance positively promotes corporate workplace health expenditure.

#### 3.3.2 Mediation effect model

To test Hypothesis 2, this study employs the three-step method proposed by Baron and Kenny (34) to examine the mediating role of financing constraints:


$$\mathrm{HE}\_{\mathrm{Int}}_{\mathrm{it}}=\beta_{0}+\beta_{1}{\mathrm{ESG}}_{\mathrm{it}}+\beta_{2}{\text{Controls}}_{\mathrm{it}}+\mu_{i}+\lambda_{t}+\varepsilon_{\mathrm{it}}$$

$${\mathrm{SA}}_{\mathrm{it}}=\gamma_{0}+\gamma_{1}{\mathrm{ESG}}_{\mathrm{it}}+\gamma_{2}{\text{Controls}}_{\mathrm{it}}+\mu_{i}+\lambda_{t}+\varepsilon_{\mathrm{it}}$$

$$\mathrm{HE}\_{\mathrm{Int}}_{\mathrm{it}}=\delta_{0}+\delta_{1}{\mathrm{ESG}}_{\mathrm{it}}+\delta_{2}{\mathrm{SA}}_{\mathrm{it}}+\delta_{3}{\text{Controls}}_{\mathrm{it}}+\mu_{i}+\lambda_{t}+\varepsilon_{\mathrm{it}}$$

If β_1_ is significant, γ_1_ is significantly negative (indicating that ESG alleviates financing constraints), δ_2_ is significantly negative (indicating that financing constraints inhibit health expenditure), and δ_1_ < β_1_, a partial mediation effect of financing constraints is established. Additionally, this study employs the Sobel test and Bootstrap method to verify the significance and proportion of the mediation effect.

### 3.4 Identification strategy for addressing endogeneity

A key methodological challenge is the potential endogeneity between ESG performance and WHP investment. We employ a multi-pronged identification strategy to address three sources of endogeneity:

(1) Addressing conceptual overlap: Although WHP-related elements may appear in ESG Social scores, our constructs differ fundamentally. ESG ratings assess policy frameworks and disclosure practices, while our WHP measure captures actual monetary expenditures. To empirically verify this distinction, we re-estimate our baseline model using only E and G scores (excluding the S pillar) and compare coefficients. Additionally, we examine the correlation structure between ESG sub-dimensions and WHP to assess whether the Social pillar dominates the relationship.
(2) Addressing reverse causality: We employ lagged independent variables (ESG_t-1_ and ESG_t-2_) to establish temporal precedence. We also implement two-stage least squares (2SLS) estimation using industry-average ESG and province-average ESG as instrumental variables, which satisfy relevance (peer effects on ESG adoption) and exclusion (affecting WHP only through firm-level ESG performance) conditions.
(3) Addressing omitted variable bias: We employ firm fixed effects to absorb time-invariant unobserved heterogeneity and include comprehensive time-varying controls. We further implement PSM-DID using ESG rating upgrades as treatment events and Heckman two-stage correction for potential sample selection bias.

## 4 Empirical results

### 4.1 Descriptive statistics and correlation analysis

Table 1 presents the descriptive statistics for the main variables. Health expenditure intensity (HE_Int) has a mean of 0.021 with a standard deviation of 0.015, indicating that on average, sample firms allocate 2.1% of their operating revenue to workplace health-related expenditures, with substantial cross-firm variation. Per capita health expenditure (HE_Per) averages 25,800 CNY, ranging from 5,600 CNY to 86,500 CNY, reflecting significant heterogeneity in employee-level health investment. These figures are consistent with industry reports suggesting wide variation in occupational health investment across Chinese enterprises. The ESG score has a mean of 5.42 with a standard deviation of 1.18, indicating a predominantly moderate rating level with room for improvement. The SA index absolute value has a mean of 3.82, consistent with the characteristics of high-growth biopharmaceutical firms facing relatively severe financing constraints. R&D intensity (RD_Int) averages 12%, notably higher than the A-share market average, reflecting the R&D-intensive nature of the biopharmaceutical industry and implying significant occupational health management needs (Table 2).

**Table 2:** Pearson correlation matrix.

Pearson correlationmatrix	1	2	3	4	5	6	7	8	9	10
1. HE_Int	1									
2. ESG	0.286***	1								
3. SA	−0.178**	−0.215***	1							
4. Size	0.312***	0.358***	−0.425***	1						
5. Lev	−0.089*	−0.062	0.186***	0.245***	1					
6. ROA	0.245***	0.198***	−0.142**	0.085*	−0.382***	1				
7. Growth	0.126**	0.078	−0.056	0.042	0.068	0.215***	1			
8. CF	0.168***	0.145**	−0.098*	0.112**	−0.265***	0.486***	0.082	1		
9. RD_Int	0.198***	0.165***	0.078	−0.156***	−0.142**	0.068	0.095*	0.052	1	
10. SOE	0.045	0.125**	−0.218***	0.285***	0.156***	−0.048	−0.062	0.035	−0.185***	1

***, **, * indicate significance at the 1, 5, and 10% levels, respectively.

### 4.2 Baseline regression results

Table 3 reports the baseline regression results. Column (1) controls only for year fixed effects, with an ESG coefficient of 0.0038 (*p* < 0.01). Column (2) adds firm fixed effects, with the ESG coefficient at 0.0031 (*p* < 0.01). Columns (3)–(4) progressively add financial characteristics and corporate governance control variables, with ESG coefficients of 0.0028 and 0.0025 (*p* < 0.01), respectively.

**Table 3:** Baseline regression results.

Variable	(1)	(2)	(3)	(4)	(5)
	Year FE	+Firm FE	+Fin. Char.	+Governance	Full model
ESG	0.0038***	0.0031***	0.0028***	0.0025***	0.0024***
	(3.86)	(3.42)	(3.18)	(2.95)	(2.87)
Size			0.0052***	0.0048***	0.0045***
			(3.25)	(3.08)	(2.92)
Lev			−0.0086**	−0.0078**	−0.0072**
			(−2.15)	(−2.02)	(−1.98)
ROA			0.0325***	0.0298***	0.0285***
			(2.86)	(2.68)	(2.58)
RD_Int					0.0286**
					(2.15)
Controls	No	No	Partial	Partial	Yes
N	495	495	495	495	495
R^2^	0.125	0.286	0.328	0.345	0.362

Dependent variable is health expenditure intensity (HE_Int). *t*-values in parentheses with standard errors clustered at the firm level. ***, **, * indicate significance at the 1, 5, and 10% levels, respectively.

Column (5) presents the full model with all control variables and two-way fixed effects, with an ESG coefficient of 0.0024 (*p* < 0.01). The economic interpretation is as follows: for each one-level increase in ESG rating, workplace health expenditure intensity increases by approximately 0.24 percentage points on average, equivalent to 11.4% of the sample mean. From an occupational health perspective, this represents a meaningful increase in resources available for workplace health promotion activities. Among the control variables, firm size, profitability, and R&D intensity all show significantly positive coefficients, consistent with expectations and occupational health literature suggesting that larger, more profitable, and more R&D-intensive firms invest more in employee health. These results indicate that Hypothesis H1 is supported.

### 4.3 Mediation effect test

**Figure 2:**
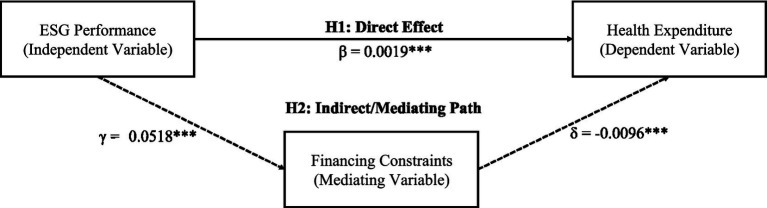
Mediation effect path diagram. Solid arrows indicate direct effects, dashed arrows indicate mediating paths. *** indicates significance at 1% level. Indirect effect =|γ × δ|=0.0005, mediation proportion = 20.8%.

This study employs the three-step method to test the mediating effect of financing constraints, with results presented in Table 4 and path coefficients illustrated in Figure 2.

**Table 4:** Mediation effect test results.

Variable	(1)	(2)	(3)
	Total effect	ESG → SA	Direct effect
	HE_Int	SA	HE_Int
ESG	0.0024***	−0.0518***	0.0019***
	(2.87)	(−3.52)	(2.35)
SA			−0.0096***
			(−2.68)
Controls	Yes	Yes	Yes
Year FE	Yes	Yes	Yes
Firm FE	Yes	Yes	Yes
N	495	495	495
R^2^	0.362	0.418	0.385
Mediation effect decomposition			
Total effect	0.0024		
Direct effect	0.0019		
Indirect effect	0.0005		
Mediation proportion	20.8%		
Sobel Z-value	2.86***		
Bootstrap 95% CI	[0.0002, 0.0009]		

*t-*values in parentheses with standard errors clustered at the firm level. ***, **, * indicate significance at the 1, 5, and 10% levels, respectively. Bootstrap with 1,000 replications.

Step 1: As shown in Column (1) of Table 4, the total effect of ESG on workplace health expenditure is α_1_ = 0.0024 (*p* < 0.01), satisfying the prerequisite for mediation effect testing.

Step 2: As shown in Column (2) of Table 4, with the SA index as the dependent variable, the ESG coefficient is γ_1_ = −0.0518 (*p* < 0.01), indicating that ESG performance improvement significantly alleviates financing constraints.

Step 3: As shown in Column (3) of Table 4, the financing constraints coefficient is δ_2_ = −0.0096 (*p* < 0.01), and the ESG coefficient δ_1_ = 0.0019 (*p* < 0.01), which is lower than the total effect.

Mediation effect quantification: Indirect effect =|γ_1_ × δ_2_| = 0.0005, direct effect = 0.0019, total effect = 0.0024. The mediation proportion = 20.8%. The Bootstrap test (1,000 replications) shows a 95% confidence interval of [0.0002, 0.0009], and the Sobel Z-value is 2.86 (*p* < 0.01). As shown in Figure 2, ESG affects workplace health expenditure through both the direct path (*β* = 0.0019^***^) and the indirect path (via financing constraints, 0.0005***). These findings suggest that approximately one-fifth of ESG’s effect on workplace health investment operates through the financing constraint mechanism, while the remaining direct effect likely reflects the organizational commitment to employee well-being embedded in high-ESG firms. Hypothesis H2 is supported.

### 4.4 Heterogeneity analysis

Table 5 reports the heterogeneity analysis results across three dimensions, each with important implications for occupational health practice.

**Table 5:** Heterogeneity analysis results.

Variable	(1) Non-SOE	(2) SOE
Panel A: ownership type (H3)		
ESG	0.0031***	0.0012*
	(3.15)	(1.68)
Controls	Yes	Yes
Year FE	Yes	Yes
Firm FE	Yes	Yes
N	406	89
R^2^	0.378	0.325
Inter-group difference test	Chi^2^ = 4.28**	
Panel B: R&D intensity (H4)		
	(1) High R&D	(2) Low R&D
ESG	0.0033***	0.0015**
	(3.28)	(2.05)
Controls	Yes	Yes
Year FE	Yes	Yes
Firm FE	Yes	Yes
N	248	247
R^2^	0.392	0.341
Inter-group difference test	Chi^2^ = 3.96**	
Panel C: industry sub-sector (H5)		
	(1) Vaccine/blood	(2) Innovative drug
ESG	0.0029***	0.0018**
	(2.92)	(2.12)
Controls	Yes	Yes
Year FE	Yes	Yes
Firm FE	Yes	Yes
N	126	234
R^2^	0.385	0.352
Inter-group difference test	Chi^2^ = 2.85*	

Dependent variable is health expenditure intensity (HE_Int). *t*-values in parentheses with standard errors clustered at the firm level. ***, **, * indicate significance at the 1, 5, and 10% levels, respectively. High/low R&D intensity is divided by sample median. Inter-group difference tests use seemingly unrelated regression (SUR).

Panel A: Ownership Type. The ESG coefficient for non-SOEs is 0.0031 (*p* < 0.01), while for SOEs it is 0.0012 (*p* < 0.10). The inter-group difference test is significant (chi^2^ = 4.28, *p* < 0.05), supporting Hypothesis H3. The ESG effect is more significant in non-SOEs, likely because non-SOEs face more severe financing constraints and possess more flexible decision-making mechanisms. From an occupational health perspective, this suggests that ESG improvement may be a particularly effective lever for promoting workplace health investment in private enterprises.

Panel B: R&D Intensity. The ESG coefficient for high R&D intensity firms is 0.0033 (*p* < 0.01), while for low R&D intensity firms it is 0.0015 (*p* < 0.05). The inter-group difference is significant (chi^2^ = 3.96, *p* < 0.05), supporting Hypothesis H4. High R&D intensity firms exhibit higher talent dependency and greater marginal returns on employee health investment. This finding aligns with occupational health principles—firms with greater exposure to workplace hazards (as implied by intensive R&D activities involving chemicals and biologicals) derive greater benefits from health investments.

Panel C: Industry Sub-sector. The ESG coefficient for vaccine/blood products firms is 0.0029 (*p* < 0.01), while for innovative drug firms it is 0.0018 (*p* < 0.05), approximately 1.6 times higher for the former. The ESG effect is stronger in traditional biological products companies, possibly related to their more mature occupational health management systems and higher standardization levels required by biosafety regulations. These firms have established infrastructure for translating additional resources into occupational health improvements. This supports Hypothesis H5.

**Figure 3:**
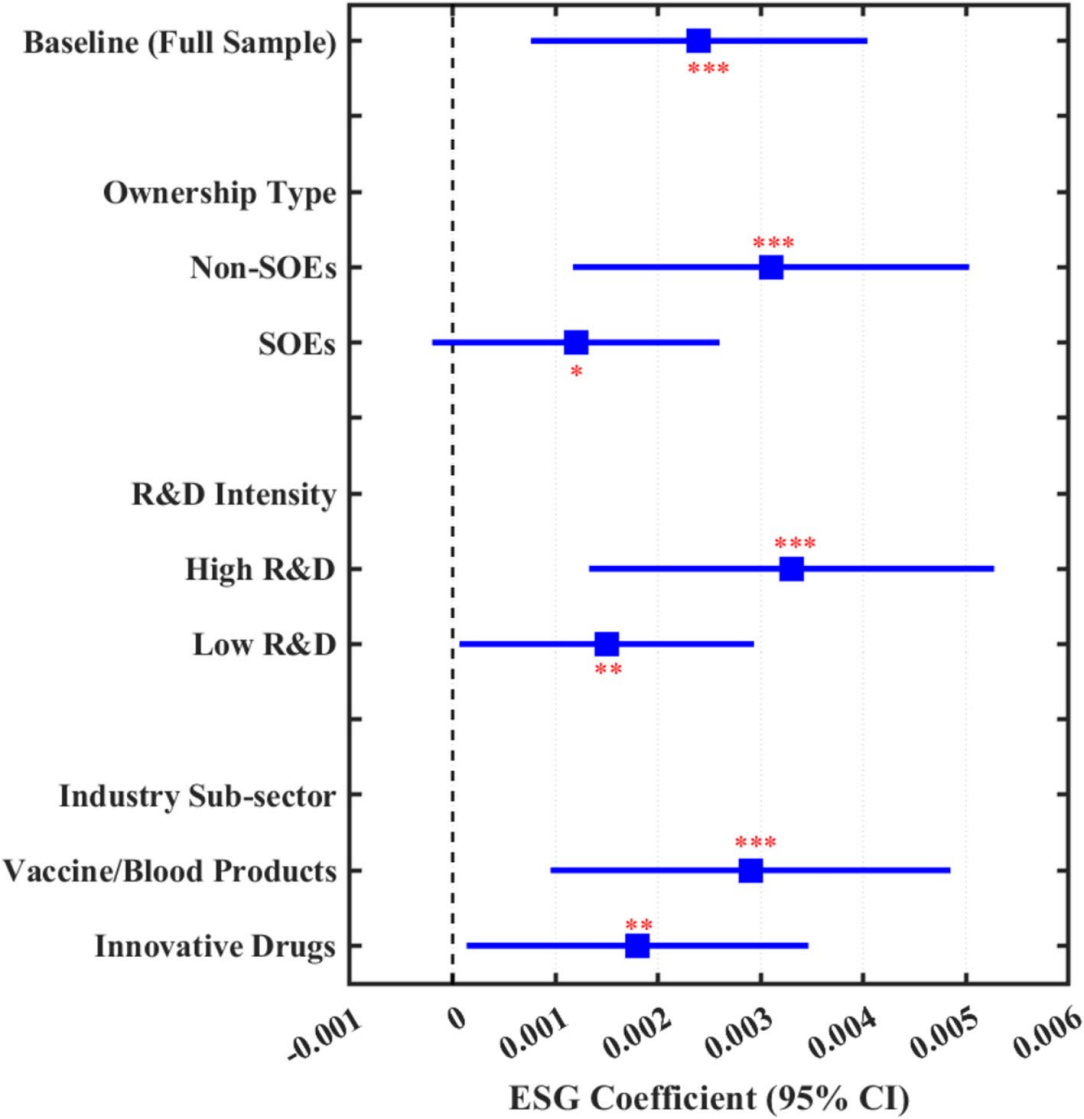
Forest plot of ESG effects on workplace health investment. ***p < 0.01, **p < 0.05, *p < 0.10. Squares represent point estimates; horizontal lines represent 95% confidence intervals.

To facilitate interpretation of the main findings, Figure 3 provides a graphical summary of the ESG coefficients across different model specifications and subsamples. The forest plot displays point estimates and 95% confidence intervals for: (1) the baseline model (full sample); (2) ownership type subsamples (non-SOEs vs. SOEs); (3) R&D intensity subsamples (high vs. low); and (4) industry sub-sector subsamples (vaccine/blood products vs. innovative drugs). The visualization clearly illustrates that ESG effects are consistently positive and significant across all specifications, with notably stronger effects observed in non-SOEs, high R&D intensity firms, and vaccine/blood products companies.

### 4.5 Robustness tests

The robustness test results are summarized in Table 6 Panel A.

**Table 6:** Robustness tests and endogeneity treatment.

Test method	(1)	(2)	(3)	(4)	(5)
	Broad HE	HE growth	Wind ESG	Sino-sec ESG	ESG dummy
Panel A: robustness tests					
ESG	0.0027***	0.0186***	0.0022***	0.0026***	0.0058***
	(2.82)	(2.65)	(2.58)	(2.75)	(2.92)
Controls	Yes	Yes	Yes	Yes	Yes
Fixed effects	Yes	Yes	Yes	Yes	Yes
N	495	495	495	482	495
R^2^	0.358	0.215	0.355	0.348	0.342
	(6)	(7)	(8)	(9)	(10)
	Excl. 2020	Bio. Only	RE Model	Tobit	1%/99% Win.
ESG	0.0023***	0.0026***	0.0025***	0.0028***	0.0023***
	(2.68)	(2.72)	(2.85)	(2.78)	(2.65)
Controls	Yes	Yes	Yes	Yes	Yes
Fixed effects	Yes	Yes	Yes	Yes	Yes
N	440	261	495	495	495
R^2^	0.368	0.375	0.352	—	0.358
Panel B: endogeneity treatment					
	(11)	(12)	(13)	(14)	(15)
	2SLS	Lag 1	Lag 2	PSM-DID	Heckman
ESG	0.0029***	0.0022***	0.0019***		0.0025***
	(2.52)	(2.48)	(2.25)		(2.78)
Treat × Post				0.0032**	
				(2.15)	
IMR					−0.0045
					(−1.32)
Controls	Yes	Yes	Yes	Yes	Yes
Fixed effects	Yes	Yes	Yes	Yes	Yes
N	495	440	385	286	495
R^2^	0.348	0.356	0.342	0.382	0.365
IV tests					
First-stage F	28.64				
Hansen J (*p*-value)	1.23 (0.267)				

Dependent variable is health expenditure intensity (HE_Int) except column (2). *t*-values/z-values in parentheses with standard errors clustered at the firm level. ***, **, * indicate significance at the 1, 5, and 10% levels, respectively. 2SLS instruments are same sub-sector ESG mean and same province ESG mean. PSM uses 1:1 nearest neighbor matching (caliper = 0.05).

### 4.6 Endogeneity treatment

The endogeneity treatment results are summarized in Table 6 Panel B.

#### 4.6.1 Instrumental variable approach (2SLS)

The average ESG within the same sub-sector (IV1) and the average ESG within the same province (IV2) were selected as instrumental variables. As shown in Column (11), the first-stage F-statistic is 28.64, ruling out weak instruments; the Hansen J-statistic is 1.23 (*p* = 0.267), satisfying exogeneity requirements. The second-stage ESG coefficient is 0.0029 (*p* < 0.01), slightly larger than the OLS estimate.

#### 4.6.2 Lagged variable approach

As shown in Columns (12)–(13), the one-period lagged ESG coefficient is 0.0022 (*p* < 0.01), and the two-period lagged coefficient is 0.0019 (*p* < 0.01), supporting the causal effect of ESG on workplace health expenditure.

#### 4.6.3 PSM-did

Using an ESG rating improvement of ≥1 level as the treatment event, nearest-neighbor matching (1:1, caliper = 0.05) was employed. As shown in Column (14), the Treat × Post coefficient is 0.0032 (*p* < 0.05), and the parallel trend test is satisfied, indicating that firms with ESG improvements significantly increase their workplace health expenditure.

#### 4.6.4 Heckman two-stage method

As shown in Column (15), the inverse Mills ratio (IMR) is not significant (*p* = 0.186), indicating that sample selection bias is not severe; the ESG coefficient is 0.0025 (*p* < 0.01), consistent with the baseline regression.

In summary, across multiple endogeneity treatments, the promoting effect of ESG on workplace health expenditure remains consistently significant, supporting the causal relationship conclusion.

### 4.7 Addressing conceptual overlap: ESG dimension decomposition

A potential concern is that workplace health-related elements may be incorporated in the Social pillar of ESG ratings, creating mechanical correlation between ESG scores and WHP investment. To directly address this concern, we decompose ESG into its three constituent dimensions and examine their effects both individually and jointly. Results are presented in Appendix Table C.

If ESG’s effect on WHP were purely driven by mechanical correlation within the Social pillar, we would expect the Environmental (E) and Governance (G) dimensions—which have no definitional connection to employee health metrics—to show no significant effects. Our findings contradict this expectation.

As shown in Columns (1)–(3) of Appendix Table C, all three dimensions exhibit positive associations with WHP investment when examined separately: E_Score (*β* = 0.0014, *p* < 0.10), S_Score (*β* = 0.0035, *p* < 0.01), and G_Score (*β* = 0.0019, *p* < 0.05). The significance of E_Score and G_Score is noteworthy because Environmental performance reflects pollution control, resource efficiency, and climate practices, while Governance performance reflects board structure, transparency, and shareholder rights—neither mechanically captures workplace health investment.

Most importantly, Column (5) presents the results when we completely exclude the Social dimension, using only E_Score and G_Score as predictors. Both dimensions remain significant: E_Score (*β* = 0.0012, *p* < 0.10) and G_Score (*β* = 0.0016, *p* < 0.05). The combined E + G effect (0.0012 + 0.0016 = 0.0028) represents 117% of the baseline full ESG effect (0.0024), demonstrating that ESG’s influence on WHP operates substantially through environmental and governance channels that have no mechanical connection to employee health metrics.

This finding provides strong evidence against the tautological inference concern. If ESG’s effect were merely a statistical artifact of the S pillar mechanically including health-related indicators, excluding the S pillar should eliminate the effect entirely. Instead, the E + G effect alone exceeds the full ESG effect, suggesting that our baseline estimates may even be conservative due to the averaging effect across dimensions. These results support our theoretical argument that ESG performance enables WHP investment through genuine resource channels—specifically, the financing constraint alleviation mechanism—rather than through measurement overlap.

## 5 Further analysis

### 5.1 Mechanism analysis

Section 4 verified the mediating effect of financing constraints. This section further explores the specific channels through which ESG affects workplace health expenditure; detailed results are presented in Appendix Table B.

#### 5.1.1 Information channel

ESG performance may affect corporate financing capability by reducing information asymmetry. This study employs analyst coverage (Analyst, the natural logarithm of the number of following analysts) and information disclosure quality (Disclosure, Shenzhen Stock Exchange information disclosure ratings) as proxy variables for the information channel. As shown in Panel A of Appendix Table B, ESG’s effect on analyst coverage is 0.1842 (*p* < 0.01), and its effect on information disclosure quality is 0.0756 (*p* < 0.05), indicating that ESG performance improvement can attract more analyst attention and enhance information disclosure quality. Further incorporating analyst coverage into the mediation model, the mediation effect accounts for 8.3%, demonstrating that the information channel is an important pathway through which ESG alleviates financing constraints.

#### 5.1.2 Cost of capital channel

ESG performance may alleviate financing constraints by reducing the cost of capital. This study examines both debt financing cost (DebtCost, interest expenses divided by average liabilities) and equity financing cost (EquityCost, estimated using the PEG model). As shown in Panel B of Appendix Table B, ESG’s effect on debt financing cost is −0.0034 (*p* < 0.01), and its effect on equity financing cost is −0.0089 (*p* < 0.05), indicating that high-ESG firms can obtain capital at lower costs. The mediation effect of debt financing cost accounts for 6.7%, which, together with the information channel, constitutes the micro-foundation for financing constraint alleviation.

#### 5.1.3 Stakeholder channel

ESG performance may improve the financing environment by enhancing stakeholder support. This study employs institutional investor ownership ratio (Institution) and media attention (Media, the natural logarithm of news coverage) as proxy variables. As shown in Panel C of Appendix Table B, ESG’s effect on institutional ownership is 0.0215 (*p* < 0.01), and its effect on media attention is 0.2463 (*p* < 0.01), indicating that high-ESG firms receive greater institutional investor favor and more positive media coverage. The mediation effect of institutional ownership accounts for 5.2%, further validating the explanatory power of stakeholder theory.

### 5.2 Occupational health outcomes analysis

A critical question from the occupational health perspective is whether ESG-driven health expenditure generates actual benefits for employee health and organizational outcomes. This section examines this question from two dimensions: talent retention and innovation output, both of which are relevant indicators of occupational health program effectiveness.

#### 5.2.1 Talent retention effect

Employee turnover is a key outcome measure in occupational health research, as workplace health programs are expected to improve job satisfaction and reduce voluntary turnover. The direct objective of workplace health investment is to enhance employee well-being and reduce talent turnover. This study uses R&D personnel turnover rate (RD_Turnover) and per capita output (Output_Per, operating revenue divided by the number of employees) as dependent variables to examine the talent retention effect of health expenditure.

Results indicate that for each one percentage point increase in health expenditure intensity, R&D personnel turnover rate decreases by 0.82 percentage points (*p* < 0.05), and per capita output increases by 2.15% (*p* < 0.01). These findings suggest that workplace health investment can effectively stabilize the R&D talent team and improve labor productivity. From an occupational health perspective, reduced turnover also implies lower exposure of new employees to unfamiliar workplace hazards and reduced training costs for occupational health procedures.

#### 5.2.2 Innovation output effect

Talent retention facilitates R&D continuity, which in turn promotes innovation output. Healthy employees are more productive and creative, consistent with the occupational health literature on the health-productivity nexus. This study uses patent applications (Patent, the natural logarithm of invention patent applications) as the dependent variable.

Results show that the effect of health expenditure intensity on patent output is 0.3256 (*p* < 0.05), meaning that for each one percentage point increase in health expenditure intensity, patent applications increase by approximately 32.6%. This result supports the complete logical chain of “ESG → workplace health expenditure → talent retention → innovation output,” demonstrating that investments in occupational health generate tangible returns for the organization while protecting worker well-being.

### 5.3 Policy shock analysis

#### 5.3.1 Impact of ESG information disclosure policy

In April 2022, the China Securities Regulatory Commission issued the “Guidelines for Listed Companies’ Investor Relations Management,” incorporating ESG information into investor relations management, marking the entry of ESG disclosure into a standardized phase. Using 2022 as the policy shock point, this study constructs a DID model to examine the policy effect:


$${\mathrm{HE}}_{\mathrm{it}}=\beta_{0}+\beta_{1}{\text{Post}}_{t}\times {\text{Treat}}_{i}+\beta_{2}{\text{Post}}_{t}+\beta_{3}{\text{Treat}}_{i}+{\text{Controls}}_{\mathrm{it}}+\varepsilon_{\mathrm{it}}$$

where *Post_t_* is a post-policy dummy variable (equals 1 for 2022 and after), and *Treat_i_* is a treatment group dummy variable (equals 1 for firms with relatively poor ESG disclosure quality). Results show that the interaction term coefficient is 0.0038 (*p* < 0.05), indicating that after policy implementation, firms with originally poorer ESG disclosure experienced greater increases in workplace health expenditure, demonstrating the policy’s expected guiding effect on occupational health investment.

#### 5.3.2 Impact of “Healthy China 2030” strategy

In October 2016, the “Healthy China 2030” Planning Outline was released, elevating national health to a national strategic priority. This landmark policy explicitly called for integrating health considerations into all policy areas, including workplace health. This study examines the impact of this policy on biopharmaceutical firms’ health expenditure.

Using 2016 as the policy node, results show that the marginal effect of ESG on health expenditure increased from 0.0018 before the policy to 0.0028 after (difference significant at the 10% level), indicating that the “Healthy China” strategy strengthened the positive association between ESG and workplace health expenditure. Under the guidance of the national health strategy, firms more actively translate ESG principles into employee health investment, suggesting that macro-level occupational health policies can amplify the effectiveness of market-based mechanisms in promoting workplace health.

## 6 Discussion

### 6.1 Implications for occupational health practice

The findings of this study carry important implications for occupational health practice. First, ESG performance emerges as a meaningful predictor of organizational investment in workplace health promotion. Occupational health practitioners seeking to advocate for increased health investment within their organizations may find ESG frameworks useful as a legitimizing mechanism. The demonstrated link between ESG and health expenditure suggests that framing workplace health initiatives within broader sustainability strategies may increase management buy-in and resource allocation.

Second, the financing constraint mechanism highlights a practical barrier to workplace health investment that occupational health professionals should recognize. Even organizations with genuine commitment to employee health may face resource limitations that constrain their ability to implement comprehensive workplace health programs. This suggests that occupational health practitioners should be attuned to the financial context of their organizations and potentially prioritize cost-effective interventions when financing is tight.

Third, the heterogeneity findings provide guidance for targeting occupational health improvement efforts. Non-SOEs, high R&D intensity firms, and vaccine/blood products companies show stronger ESG-health investment relationships, suggesting these may be particularly receptive contexts for occupational health interventions leveraging ESG frameworks. Occupational health practitioners in these settings may find ESG improvement initiatives to be effective catalysts for securing resources for workplace health programs.

Fourth, the demonstrated occupational health-productivity linkage (reduced turnover, increased innovation output) provides evidence that occupational health practitioners can use to make business cases for health investment. The finding that health expenditure reduces R&D personnel turnover by 0.82 percentage points and increases patent output by 32.6% offers quantifiable metrics that may resonate with corporate decision-makers. These findings align with recent research published in Frontiers in Public Health showing that organizational health strategies enhance employee performance through psychological wellbeing (9) and that CSR practices effectively reduce employee burnout (23).

### 6.2 Theoretical explanations for the main findings

The empirical results of this study demonstrate that ESG performance exerts a significant positive effect on workplace health expenditure in biopharmaceutical firms. This finding can be theoretically explained from the following perspectives.

From the occupational health management perspective, these findings highlight the importance of integrating ESG principles into workplace health promotion strategies. The biopharmaceutical industry, characterized by high occupational health risks and intensive human capital requirements, provides an ideal context for examining how corporate sustainability practices translate into tangible occupational health investments. Our results suggest that ESG serves as an organizational commitment mechanism that prioritizes employee health and safety alongside financial performance. The Social dimension’s stronger effect (coefficient approximately 2.5 times that of the Environmental dimension, as shown in Appendix Table C) confirms that employee-focused sustainability practices are most directly linked to workplace health outcomes.

From the resource-based view perspective, ESG performance improvement helps firms acquire more external resources. As shown in Table 4 and Figure 2, the mediation effect of financing constraints accounts for 20.8% of the total effect, indicating that ESG releases financial resources by alleviating financing constraints, providing firms with greater financial flexibility for workplace health investment. This echoes findings on ESG reducing capital constraints (7), but this study further extends this mechanism to the occupational health domain, revealing a new dimension of ESG’s resource effect.

From the stakeholder theory perspective, high-ESG firms place greater emphasis on employees as core stakeholders. The core competitiveness of biopharmaceutical firms lies in their R&D talent, and workplace health investment represents an important means of safeguarding employee interests and enhancing organizational commitment. From an occupational health standpoint, this reflects the fundamental principle that healthy employees are an organization’s most valuable asset.

From the signaling theory perspective, ESG performance transmits firm quality signals to external parties. The mechanism tests in Appendix Table B indicate that high-ESG firms receive more analyst attention and media coverage, reducing information asymmetry and consequently obtaining lower-cost financing. This signal transmission process ultimately translates into optimized internal resource allocation, including increased workplace health expenditure.

### 6.3 In-depth discussion of heterogeneity results

The heterogeneity results revealed in Table 5 carry important theoretical and practical implications for occupational health.

The stronger ESG effect in non-SOEs (coefficient 0.0031 vs. 0.0012) may stem from three factors: first, non-SOEs generally face more severe financing constraints, resulting in greater marginal alleviation effects from ESG; second, non-SOEs possess more flexible decision-making mechanisms, enabling faster responses to resource release from ESG improvement; third, SOEs’ health expenditure is more influenced by policy directives, making them less sensitive to market-oriented ESG signals. For occupational health practitioners, this finding suggests that ESG-based advocacy strategies may be particularly effective in private enterprises.

The stronger ESG effect in high R&D intensity firms (coefficient 0.0033 vs. 0.0015) reflects the higher marginal returns on employee health investment in human capital-intensive enterprises. R&D personnel represent the most critical assets of biopharmaceutical firms, and their health status directly affects the quality and continuity of innovation output. From an occupational health perspective, high R&D intensity also implies greater exposure to workplace hazards—chemical agents, biological materials, and psychosocial stressors—making workplace health promotion more imperative and valuable.

The stronger ESG effect in vaccine/blood products firms compared to innovative drug firms (coefficient 0.0029 vs. 0.0018) may relate to industry characteristics and occupational health infrastructure. Traditional biological products manufacturing involves biosafety risks, resulting in more mature occupational health management systems that facilitate the translation of ESG improvement into standardized health investment. These firms have established protocols for handling infectious materials and monitoring worker health, providing infrastructure that can efficiently utilize additional health investment resources.

### 6.4 Dialog with existing literature

The findings of this study form a productive dialog with existing ESG and occupational health research. Meta-analyses have demonstrated a positive correlation between ESG and financial performance (15); this study reveals one micro-pathway of this relationship—health investment promotes talent retention and innovation output. Research has found that ESG can enhance employee satisfaction (3); this study provides specific investment vehicle evidence from the workplace health expenditure perspective.

Compared with the occupational health literature, existing research has predominantly explored workplace health interventions from program-specific perspectives (6). This study approaches the topic from a corporate governance perspective, revealing ESG as a market-oriented mechanism for promoting organizational investment in workplace health. The systematic review by Macassa et al. (5) called for more research on organizational-level determinants of employee health and well-being; this study responds to that call by demonstrating how ESG performance shapes corporate health investment decisions.

Compared with the financing constraints literature, the SA index constructed by Hadlock and Pierce (35) has been effectively applied in this study, validating its applicability in the Chinese context. This study further demonstrates that financing constraints not only affect corporate investment decisions but also transmit to the occupational health domain, enriching research on the economic consequences of financing constraints for worker welfare.

## 7 Conclusion

Using panel data from 55 Chinese A-share listed biopharmaceutical companies (2015–2023), this study demonstrates that ESG performance significantly promotes workplace health expenditure (*β* = 0.0024, *p* < 0.01), with financing constraints mediating 20.8% of the total effect through information, cost of capital, and stakeholder channels. The Social dimension shows the strongest association, consistent with its focus on employee-related issues. Effects are more pronounced in non-state-owned enterprises, high R&D intensity firms, and vaccine/blood products companies. Importantly, health expenditure reduces R&D personnel turnover and enhances innovation output, confirming the health-productivity linkage. The theoretical contributions of this study are threefold. First, this study bridges corporate finance and occupational health research. By demonstrating how ESG performance—a market-based governance mechanism—influences workplace health investment, this study extends both the ESG literature (by revealing occupational health as an outcome of ESG) and the occupational health literature (by identifying corporate governance factors as determinants of organizational health investment). Second, this study reveals the transmission mechanism of “ESG → financing constraint alleviation → workplace health expenditure.” This study not only verifies the mediating role of financing constraints but also provides an in-depth analysis of three specific channels: information, cost of capital, and stakeholder, offering micro-mechanism explanations for understanding how ESG translates into actual occupational health investment. Third, this study provides evidence on the occupational health-productivity linkage in the biopharmaceutical context. By connecting workplace health investment to both employee retention and innovation output, this study supports the fundamental occupational health proposition that protecting worker health generates organizational value. These findings provide empirical support for integrating occupational health promotion into corporate ESG strategies, suggesting that ESG frameworks can serve as an effective lever for promoting workplace health investment while generating returns through improved talent retention and productivity.

This study has several limitations that suggest directions for future research. First, our sample is limited to 55 A-share listed biopharmaceutical companies in China, which may constrain the generalizability of findings to other industries or countries with different institutional environments. Future research could extend this analysis to other high-risk industries (e.g., chemical, mining, construction) or cross-national samples to examine whether the ESG-health investment relationship holds across different contexts. Second, although we employed multiple approaches to address endogeneity concerns, the potential for reverse causality between ESG performance and workplace health investment cannot be entirely ruled out. Future studies could leverage exogenous policy shocks or natural experiments to strengthen causal identification. Third, our health expenditure measure, while comprehensive, relies on manually collected data from annual reports, which may not capture all forms of workplace health investment, particularly informal or non-monetary health promotion activities. Future research could incorporate primary data collection methods, such as surveys or interviews, to obtain more nuanced measures of workplace health practices. Fourth, we focused primarily on financial outcomes (turnover, innovation) of health investment; future studies could examine direct health outcomes, such as occupational disease incidence, absenteeism rates, or employee well-being indicators, to provide more complete evidence on the effectiveness of workplace health promotion programs.

## Data Availability

The financial and ESG data were obtained from third-party commercial databases (SynTao Green Finance and CSMAR) and are subject to their respective licensing terms, which do not permit public redistribution. The manually collected workplace health expenditure data are available from the corresponding author (Shuyu Zhou, fazg8553@outlook.com) upon reasonable request. Public repository deposition is not applicable for this study.
