# Supplementary material for: ESG performance, financing constraints, and workplace health investment: evidence from Chinese biopharmaceutical enterprises
Source: Front Public Health. 2026 Mar 9;14:1765422. doi: 10.3389/fpubh.2026.1765422 (PMC13006613; doi:10.3389/fpubh.2026.1765422)
Supplement: Supplementary file 1 [file Table_1.docx]

**APPENDIX**

**Corporate ESG Performance and Workplace Health Promotion Investment: Evidence from Chinese Biopharmaceutical Enterprises**

# Appendix Table A. Sample Firm List

| **No.** | **Stock Code** | **Abbrev.** | **Full Name** | **Sub-sector** | **Main Products** |
| --- | --- | --- | --- | --- | --- |
| 1 | 300122.SZ | Zhifei Bio | Chongqing Zhifei Biological Products Co., Ltd. | Vaccine | Vaccine leader |
| 2 | 603392.SH | Wantai Bio | Beijing Wantai Biological Pharmacy Enterprise Co., Ltd. | Vaccine | HPV vaccine |
| 3 | 300142.SZ | Walvax Bio | Yunnan Walvax Biotechnology Co., Ltd. | Vaccine | Vaccine R&D |
| 4 | 300601.SZ | Kangtai Bio | Shenzhen Kangtai Biological Products Co., Ltd. | Vaccine | Vaccine leader |
| 5 | 688739.SH | Chengda Bio | Liaoning Chengda Biotechnology Co., Ltd. | Vaccine | Rabies vaccine |
| 6 | 688185.SH | CanSino Bio | CanSino Biologics Inc. | Vaccine | COVID/meningitis |
| 7 | 688319.SH | Olymvax Bio | Chengdu Olymvax Biopharmaceuticals Inc. | Vaccine | Tetanus vaccine |
| 8 | 300841.SZ | Kanghua Bio | Chengdu Kanghua Biological Products Co., Ltd. | Vaccine | Rabies vaccine |
| 9 | 688276.SH | Changchun BCHT | Changchun BCHT Biotechnology Co., Ltd. | Vaccine | Varicella/shingles |
| 10 | 600161.SH | Tiantan Bio | Beijing Tiantan Biological Products Co., Ltd. | Blood products | Leader (SOE) |
| 11 | 002252.SZ | Shanghai RAAS | Shanghai RAAS Blood Products Co., Ltd. | Blood products | Leader |
| 12 | 002007.SZ | Hualan Bio | Hualan Biological Engineering Inc. | Blood products | Blood+vaccine |
| 13 | 000403.SZ | Pailin Bio | Paisi Shuanglin Biopharmaceutical Co., Ltd. | Blood products | Blood products |
| 14 | 300294.SZ | Boya Bio | Boya Bio-Pharmaceutical Group Co., Ltd. | Blood products | Blood products |
| 15 | 002880.SZ | Weiguang Bio | Shenzhen Weiguang Biological Products Co., Ltd. | Blood products | Blood products |
| 16 | 000661.SZ | Changchun High-Tech | Changchun High & New Technology Industry (Group) Inc. | Biologics | GH leader |
| 17 | 603087.SH | Gan & Lee | Gan & Lee Pharmaceuticals Co., Ltd. | Biologics | Insulin |
| 18 | 600867.SH | Tonghua Dongbao | Tonghua Dongbao Pharmaceutical Co., Ltd. | Biologics | Insulin |
| 19 | 300009.SZ | Anke Bio | Anhui Anke Biotechnology (Group) Co., Ltd. | Biologics | GH/interferon |
| 20 | 688278.SH | Amoytop Bio | Xiamen Amoytop Biotech Co., Ltd. | Biologics | Interferon |
| 21 | 300357.SZ | Wolwo Bio | Zhejiang Wolwo Bio-Pharmaceutical Co., Ltd. | Biologics | Allergen immunotherapy |
| 22 | 002581.SZ | Weiming Medicine | Shandong Weiming Biomedical Co., Ltd. | Biologics | Interferon |
| 23 | 300683.SZ | Hisun Bio | Wuhan Hiteck Biological Pharma Co., Ltd. | Biologics | NGF |
| 24 | 600211.SH | Tibet Pharma | Tibet Rhodiola Pharmaceutical Holding Co., Ltd. | Biologics | Peptides |
| 25 | 000518.SZ | Sihuan Bio | Jiangsu Sihuan Bioengineering Co., Ltd. | Biologics | Cytokines |
| 26 | 300653.SZ | Zhenghai Bio | Yantai Zhenghai Bio-Tech Co., Ltd. | Biologics | Regenerative medicine |
| 27 | 688068.SH | Hotgen Bio | Beijing Hotgen Biotech Co., Ltd. | Biologics | Diagnostics |
| 28 | 300318.SZ | Bohui Innovation | Beijing Bohui Innovation Biotech Group Co., Ltd. | Biologics | Diagnostics+blood |
| 29 | 688180.SH | Junshi Bio | Shanghai Junshi Biosciences Co., Ltd. | Biologics/mAb | PD-1 mAb |
| 30 | 688331.SH | RemeGen | RemeGen Co., Ltd. | Biologics/mAb | ADC/mAb |
| 31 | 688062.SH | Mabwell Bio | Mabwell (Shanghai) Bioscience Co., Ltd. | Biologics/mAb | mAb |
| 32 | 688336.SH | Sunshine Guojian | Sunshine Guojian Pharmaceutical (Shanghai) Co., Ltd. | Biologics/mAb | mAb |
| 33 | 688520.SH | Sinocelltech | Beijing Sinocelltech Group Co., Ltd. | Biologics/mAb | Hemophilia/mAb |
| 34 | 688221.SH | Frontier Bio | Frontier Biotechnologies Inc. | Innovative drug | Anti-HIV |
| 35 | 600276.SH | Hengrui Medicine | Jiangsu Hengrui Pharmaceuticals Co., Ltd. | Innovative drug | Leader |
| 36 | 688235.SH | BeiGene | BeiGene, Ltd. | Innovative drug | BTK/PD-1 |
| 37 | 600196.SH | Fosun Pharma | Shanghai Fosun Pharmaceutical (Group) Co., Ltd. | Innovative drug | Diversified |
| 38 | 300558.SZ | Betta Pharma | Betta Pharmaceuticals Co., Ltd. | Innovative drug | EGFR/ALK inhibitor |
| 39 | 002422.SZ | Kelun Pharma | Sichuan Kelun Pharmaceutical Co., Ltd. | Innovative drug | ADC/innovative |
| 40 | 000963.SZ | Huadong Medicine | Huadong Medicine Co., Ltd. | Innovative drug | GLP-1/autoimmune |
| 41 | 600079.SH | Humanwell Healthcare | Humanwell Healthcare (Group) Co., Ltd. | Innovative drug | Anesthesia/analgesic |
| 42 | 688578.SH | Aelis Pharma | Shanghai Aelis Pharmaceuticals Co., Ltd. | Innovative drug | 3rd-gen EGFR |
| 43 | 688506.SH | Baili-Tianheng | Sichuan Baili Pharmaceutical Co., Ltd. | Innovative drug | ADC |
| 44 | 300199.SZ | Hybio Pharma | Shenzhen Hybio Pharmaceutical Co., Ltd. | Innovative drug | Peptide drugs |
| 45 | 002399.SZ | Hepalink | Shenzhen Hepalink Pharmaceutical Group Co., Ltd. | Innovative drug | Heparin/innovative |
| 46 | 688091.SH | Shanghai Yizhong | Shanghai Yizhong Pharmaceutical Co., Ltd. | Innovative drug | Paclitaxel micelle |
| 47 | 688366.SH | Haohai Biotech | Shanghai Haohai Biological Technology Co., Ltd. | Innovative drug | Ophtha/ortho biomaterial |
| 48 | 300765.SZ | Xinuowei | CSPC Innovation Pharmaceutical Co., Ltd. | Innovative drug | API/innovative |
| 49 | 688658.SH | Yuekang Pharma | Yuekang Pharma Group Co., Ltd. | Innovative drug | Innovative drug |
| 50 | 688566.SH | Jibeier | Jiangsu Jibeier Pharmaceutical Co., Ltd. | Innovative drug | Innovative drug |
| 51 | 688176.SH | Yahong Medicine | Jiangsu Yahong Meditech Co., Ltd. | Innovative drug | Urologic oncology |
| 52 | 002603.SZ | Yiling Pharma | Shijiazhuang Yiling Pharmaceutical Co., Ltd. | TCM innovation | TCM innovative drug |
| 53 | 002317.SZ | Zhongsheng Pharma | Guangdong Zhongsheng Pharmaceutical Co., Ltd. | TCM | Compound Xueshuan |
| 54 | 300436.SZ | Guangshengtang | Fujian Guangshengtang Pharmaceutical Co., Ltd. | Chemical drug | Hepatitis B drugs |
| 55 | 603367.SH | Chenxin Pharma | Chenxin Pharmaceutical Co., Ltd. | Chemical drug | Injectables |

*Note: The sample includes 55 A-share listed biopharmaceutical companies in China, covering four sub-sectors: vaccines (8 firms), blood products (6 firms), other biological products (15 firms), and innovative drugs (26 firms). The sample period is 2015-2023. Stock code suffix SZ indicates Shenzhen Stock Exchange; SH indicates Shanghai Stock Exchange. GH = growth hormone; mAb = monoclonal antibody; ADC = antibody-drug conjugate; NGF = nerve growth factor; TCM = traditional Chinese medicine; API = active pharmaceutical ingredient.*

# Appendix Table B. Mechanism Test Results

*Panel A: Information Channel*

| **Variable** | **(1)** | **(2)** | **(3)** | **(4)** |
| --- | --- | --- | --- | --- |
|  | Analyst | Disclosure | HE_Int | HE_Int |
| **ESG** | 0.1842*** | 0.0756** | 0.0022*** | 0.0021*** |
|  | (3.56) | (2.15) | (2.68) | (2.52) |
| **Analyst** |  |  | 0.0012** |  |
|  |  |  | (2.08) |  |
| **Disclosure** |  |  |  | 0.0035* |
|  |  |  |  | (1.85) |
| **Controls** | Yes | Yes | Yes | Yes |
| **Fixed Effects** | Yes | Yes | Yes | Yes |
| **N** | 495 | 428 | 495 | 428 |
| **R^2^** | 0.425 | 0.382 | 0.368 | 0.372 |
| **Mediation proportion** |  |  | 8.3% |  |

*Panel B: Cost of Capital Channel*

| **Variable** | **(1)** | **(2)** | **(3)** | **(4)** |
| --- | --- | --- | --- | --- |
|  | DebtCost | EquityCost | HE_Int | HE_Int |
| **ESG** | -0.0034*** | -0.0089** | 0.0022*** | 0.0021*** |
|  | (-2.86) | (-2.25) | (2.65) | (2.58) |
| **DebtCost** |  |  | -0.0485** |  |
|  |  |  | (-2.12) |  |
| **EquityCost** |  |  |  | -0.0268* |
|  |  |  |  | (-1.78) |
| **Controls** | Yes | Yes | Yes | Yes |
| **Fixed Effects** | Yes | Yes | Yes | Yes |
| **N** | 495 | 465 | 495 | 465 |
| **R^2^** | 0.398 | 0.362 | 0.375 | 0.368 |
| **Mediation proportion** |  |  | 6.7% |  |

*Panel C: Stakeholder Channel*

| **Variable** | **(1)** | **(2)** | **(3)** | **(4)** |
| --- | --- | --- | --- | --- |
|  | Institution | Media | HE_Int | HE_Int |
| **ESG** | 0.0215*** | 0.2463*** | 0.0023*** | 0.0022*** |
|  | (2.92) | (3.68) | (2.72) | (2.65) |
| **Institution** |  |  | 0.0058** |  |
|  |  |  | (2.05) |  |
| **Media** |  |  |  | 0.0008* |
|  |  |  |  | (1.75) |
| **Controls** | Yes | Yes | Yes | Yes |
| **Fixed Effects** | Yes | Yes | Yes | Yes |
| **N** | 495 | 495 | 495 | 495 |
| **R^2^** | 0.408 | 0.435 | 0.372 | 0.365 |
| **Mediation proportion** |  |  | 5.2% |  |

*Note: Analyst = analyst coverage (natural logarithm of number of following analysts); Disclosure = information disclosure quality (Shenzhen Stock Exchange rating); DebtCost = cost of debt financing (interest expenses / average liabilities); EquityCost = cost of equity financing (estimated using PEG model); Institution = institutional investor ownership ratio; Media = media attention (natural logarithm of number of news articles). t-values in parentheses with standard errors clustered at the firm level. ***, **, * indicate significance at the 1%, 5%, and 10% levels, respectively.*

# Appendix Table C. ESG Sub-dimension Regression Results

| **Variable** | **(1)** | **(2)** | **(3)** | **(4)** | **(5)** |
| --- | --- | --- | --- | --- | --- |
|  | E_Score | S_Score | G_Score | All Included | E+G Only |
| **E_Score** | 0.0014* |  |  | 0.0008 | 0.0012* |
|  | (1.72) |  |  | (0.95) | (1.78) |
| **S_Score** |  | 0.0035*** |  | 0.0028*** |  |
|  |  | (3.25) |  | (2.62) |  |
| **G_Score** |  |  | 0.0019** | 0.0012 | 0.0016** |
|  |  |  | (2.08) | (1.35) | (2.05) |
| **Controls** | Yes | Yes | Yes | Yes | Yes |
| **Year FE** | Yes | Yes | Yes | Yes | Yes |
| **Firm FE** | Yes | Yes | Yes | Yes | Yes |
| **N** | 495 | 495 | 495 | 495 | 495 |
| **R^2^** | 0.338 | 0.372 | 0.352 | 0.378 | 0.356 |

*Note: Dependent variable is health expenditure intensity (HE_Int). E_Score, S_Score, and G_Score represent the Environmental, Social, and Governance dimension scores from SynTao ESG ratings, respectively.* *Column (5) presents results using only E_Score and G_Score, completely excluding the Social dimension to address concerns about conceptual overlap between the S pillar and workplace health investment. t-values in parentheses with standard errors clustered at the firm level. ***, **, * indicate significance at the 1%, 5%, and 10% levels, respectively.*
